# Neuromodulation of the right inferior frontal gyrus in bipolar disorder to target response inhibition: a proof-of-concept study

**DOI:** 10.1017/S1092852925100308

**Published:** 2025-06-27

**Authors:** Karling Luciani, Laura Schmid, Bazyl Carroll, Alexandra Sebastian, Fidel Vila-Rodriguez, Christian G. Schütz

**Affiliations:** 1 Department of Psychiatry, Faculty of Medicine, Institute of Mental Health, University of British Columbia, Vancouver, BC, Canada; 2 BC Mental Health & Substance Use Services Research Institute, PHSA, Vancouver, BC, Canada; 3 Non-Invasive Neurostimulation Therapies Laboratory, Department of Psychiatry, Faculty of Medicine, University of British Columbia, Vancouver, BC, Canada; 4 University Medical Center, Johannes Gutenberg University, Mainz, Germany; 5 Leibniz Institute for Resilience Research, Mainz, Germany; 6 School of Biomedical Engineering, University of British Columbia, Vancouver, BC, Canada; 7 Spanish Consortium of Biomedical Research in Epidemiology and Public Health (CIBERESP), Madrid, Spain; 8 Health Technology Assessment in Primary Care and Mental Health Research Group (PRISMA), Barcelona, Spain

**Keywords:** Bipolar disorder, response inhibition, right inferior frontal gyrus, transcranial magnetic stimulation, theta-burst stimulation

## Abstract

**Objective:**

Bipolar disorder (BD) affects over 1% of the population and is characterized by deficits in response inhibition. Response inhibition, a crucial component of executive functions, involves the ability to suppress or withhold a planned or ongoing response that is no longer required or appropriate in a given context. Response inhibition may be dissociated into three subcomponents: interference inhibition, action withholding, and action cancellation. These subcomponents are assessed using the hybrid response inhibition (HRI) task. Previous research has shown that inhibitory control is strongly lateralized to the right hemisphere. Specifically, the right inferior frontal gyrus (rIFG) is a key node underpinning response inhibition and might be amenable to neuromodulation using repetitive transcranial magnetic stimulation (rTMS). This proof-of-concept study aimed to investigate the effects of rTMS targeting the rIFG on response inhibition in individuals with BD and controls.

**Methods:**

We investigated HRI performance scores in individuals with BD (*n* = 12) and sex-/age-matched controls (*n* = 12) immediately before and after intermittent theta-burst stimulation (iTBS) and continuous TBS to modulate cortical excitability of the rIFG.

**Results:**

The response inhibition subcomponent “action withholding” was significantly improved in the HRI task following iTBS in the BD group. No other significant effects were observed in the results.

**Conclusions:**

Our study is the first to show that iTBS to the rIFG neuromodulated a specific subcomponent of response inhibition in BD. Further research investigating the potential therapeutic effect of neuromodulation of the rIFG in BD is warranted.

## Introduction

Bipolar disorder (BD) is a mental disorder characterized by shifts in mood and energy, with a lifetime prevalence of more than 1% in the global population.^1^^,^^2^ BD has a substantial impact on an individual’s everyday functioning and has the highest rate of suicide of all psychiatric conditions.^3^ Despite this, current treatment strategies for BD face substantial limitations.^4^ While pharmaceutical treatments, often combined with psychological interventions, are the most commonly utilized approaches for managing BD, their efficacy can be limited, and these treatments can induce substantial and sometimes serious adverse effects.^4^^,^^5^ Impulsivity, a prominent trait in BD, is associated with increased suicidality.^6^ Existing pharmaceutical treatments for BD have not been effective in reducing impulsivity,^7^ and no other treatment approaches prioritize impulsivity as a primary focus.

A central process that affects changes in brain function associated with BD is cognitive control, the ability to manage thoughts and behaviors in accordance with one’s goals.^8^ One component of cognitive control is being able to reverse decisions after they are made but before they are implemented, otherwise known as response inhibition.^9^ Individuals with BD have been found to be more impulsive because of deficits in response inhibition co-occurring with strong impulses, which lead to poor decision-making and functioning.^10^ Response inhibition may be dissociated into three subcomponents: (i) interference inhibition, (ii) action withholding, and (iii) action cancellation.^11^ All subcomponents share a common neural network in the prefrontal cortex but differ in the degree of regional involvement.^11^ Thus, they all constitute different subprocesses of response inhibition that should be considered when investigating cognitive control in different populations. These subcomponents have not yet been investigated in a BD population; research has primarily focused on response inhibition as a whole.

The regions of the ventrolateral prefrontal cortex involved in inhibitory control are strongly lateralized to the right hemisphere.^12^ In particular, the right inferior frontal gyrus (rIFG) plays a large role in stopping impulsive behaviors and is thus a potential target for augmentation of response inhibition and cognitive control.^10^ Reduced activation of the rIFG during response inhibition has been discovered in individuals with BD during both the euthymic (stable mood) and manic states compared to controls.^13^ Past studies have found cortical hyperactivations in BD in other brain regions, such as the adjacent prefrontal cortex and superior temporal gyrus, which may, in part, represent compensatory activity for the cognitive changes caused by the rIFG hypoactivations.^10^^,^^13^ Thus, treatments focused on stimulation of the rIFG may reduce the need for compensatory overactivity during response inhibition and improve behavioral outcomes.^10^

Repetitive transcranial magnetic stimulation (rTMS) is a noninvasive brain stimulation technique that induces electrical stimuli to the cortex using strong, rapidly changing magnetic fields to target specific brain regions.^14^ Theta-burst stimulation (TBS) is a patterned form of rTMS shown to produce strong and lasting neurophysiological effects with a reduced administration time.^15^ The two different patterns of TBS commonly used are intermittent TBS (iTBS), where pulses are delivered intermittently to increase cortical excitability, and continuous TBS (cTBS), where pulses are delivered continuously to reduce cortical excitability.^15^ rTMS is currently being investigated for mental disorders involving impulse control deficits, such as post-traumatic stress disorder and generalized anxiety disorder.^16^^,^^17^ In addition, a study investigating the effects of TBS of the rIFG on inhibitory control in individuals with a substance use disorder showed a significant improvement following iTBS.^18^ However, current investigations on the application of rTMS in BD remain limited,^19^ and no study to date has looked at the stimulation of the rIFG in BD to assess response inhibition.

The current proof-of-concept study explored neuromodulation of the rIFG and its effect on response inhibition in individuals with BD and controls. The three subcomponents of response inhibition were investigated and compared using a validated computerized task before and after TBS. The primary aim of the study was to assess the effect of iTBS and cTBS on response inhibition performance scores for individuals with BD. The secondary aim of the study was to identify any score differences between individuals with BD and controls for the effect of iTBS and cTBS on response inhibition.

## Methods

### Participants

The study included 12 euthymic adults aged 19–45 years with BD (I or II) and 12 age- and sex-matched controls. Before study enrollment, a phone screener was performed to prescreen eligibility. Participants were eligible to participate if they were right-handed, fluent in English, and had no history of any substance use disorder, gambling disorder, or any other mental disorder. Individuals were excluded if they did not meet the criteria for both the TBS and magnetic resonance imaging (MRI) safety screenings. BD participants required a confirmed diagnosis via the Mini-International Neuropsychiatric Interview (for Diagnostic and Statistical Manual of Mental Disorders psychiatric disorders)^20^ and had to be stable on psychotropic medications for at least 2 weeks before screening, while remaining in the euthymic state during this time. Several BD participants reported taking antipsychotic medications (quetiapine, *n* = 5; aripiprazole, *n* = 1), antidepressants (escitalopram, *n* = 3; bupropion, *n* = 2; trazodone, *n* = 1, for sleep), and mood stabilizers (lamotrigine, *n* = 3; divalproex, *n* = 2; lithium, *n* = 1). One participant also reported gidazepam, a benzodiazepine. No controls reported taking any regular medications. To be able to participate, participants were screened for substance use including alcohol at the start of each session by completing a urine toxicology test (One Step Multi-Line Screen Test Device, Nova Century Scientific, Burlington, ON) and an alcohol breathalyzer test (BACtrack S80, KHN Solutions Inc., San Francisco, CA). Nicotine and tobacco use were also assessed, and participants confirmed they had not used any nicotine or tobacco products for at least 12 hours before each session.

The study protocol was approved by the University of British Columbia Clinical Research Ethics Board and the Vancouver Coastal Health Research Institute. All participants provided written informed consent before study enrollment and ongoing verbal consent at the start of each study session.

### Procedures

All participants completed one baseline session to obtain demographic and clinical characteristics, one MRI session, and two TBS sessions. During the baseline session, participants completed demographic questionnaires and a brief training session on the computerized hybrid response inhibition (HRI) task. During Session 2, MRI brain scans were performed on the participants using a single Philips Achieva 3.0 Tesla MRI scanner to obtain an individual anatomical 3DT1 image for TBS targeting of the rIFG. Structural scans were acquired with a voxel size of 1 mm^3^, and functional MRI scans were collected during the HRI task (TR (repetition time) = 2000 ms, TE (echo time) = 30 ms, voxel size = 3 mm^3^). The participants received iTBS during Session 3 and cTBS during Session 4. The two TBS sessions were required to be a minimum of 24 hours and a maximum of 9 days apart. In Session 3, the researchers determined participants’ motor threshold in accordance with standard clinical practice.^21^ During both Sessions 3 and 4, participants completed the state anxiety section of the State–Trait Anxiety Inventory and the HRI task before receiving TBS (pre-TBS). Immediately following TBS (post-TBS), participants completed the HRI task again (within 5 min) and then completed a comfort-rating questionnaire to report any immediate adverse effects. The morning after each TBS session, participants completed a follow-up questionnaire to document any next-day side effects.

TBS was administered using the MagVenture MagPro X100 stimulator equipped with the MagVenture Cool-B65 coil (MagVenture, Farum, Denmark). The TBS dose was calculated as 90% of a participant’s resting motor threshold, falling within the range of 35–65% intensity. TBS stimulation began at 20–25% intensity to acclimate participants to the sensation and was increased to the participant’s dose throughout stimulation at each session. Stimulation parameters for the iTBS consisted of bursts of pulses in triplets at 50 Hz, repeated at 5 Hz, with inter-train pauses of 8 s for 20 trains to deliver a total of 600 pulses over 189 s, aimed at increasing cortical excitability.^15^ Stimulation parameters for the cTBS consisted of pulses in triplets, each with a frequency of 50 Hz and delivered every 0.2 s, applied continuously for 40 s, resulting in a total of 600 pulses, aimed at decreasing cortical excitability.^15^

The method of neuronavigation for locating each participant’s rIFG was based on their neuroanatomical 3DT1 brain scan obtained in the MRI session. A frameless stereotaxic system to target the specific area of interest was used. Neuronavigation proceeded using the system Localite (Version 3, www.localite.de) to position the coil for maximal field strength at the rIFG for each participant. The rIFG was specified by reverse coregistration from a stereotaxic coordinate on the standard Montreal Neurological Institute (MNI-152) template brain onto each participant’s anatomical MRI. The MNI coordinates for the rIFG subregion pars opercularis were *x*, *y*, *z* = +52, +13, +8, drawn from a prior study reporting a functional subdivision of Broca’s area and confirmed with the Yale BioImage MNI to Talairach Converter Tool.^22^^,^^23^ For all participants, the coil handle was oriented posteriorly (pointing backwards) and positioned tangentially to the scalp, perpendicular to the sagittal plane (ie, to an imaginary line running from the top of the head to the feet), to optimize stimulation perpendicular to the rIFG.

### Measures

The HRI task is a newly developed, validated behavioral paradigm that assesses the three subcomponents of response inhibition.^11^ In our study, the HRI task was conducted by replicating the design created by Sebastian et al.,^11^ and programmed in the software Presentation (version 16.4, www.neurobs.com). The task consisted of four conditions: a congruent go condition (62.5%), an incongruent go condition (12.5%), a no-go condition (12.5%), and a stop condition (12.5%). The total duration of the HRI task took between 8 and 9 min to complete.

Interference inhibition is defined as the ability to suppress interfering response tendencies.^24^ Interference inhibition was measured by the Simon interference effect in the HRI task. The Simon interference effect was calculated by subtracting the mean reaction time (RT) of congruent trials from the mean RT of incongruent trials.

Action withholding is defined as the ability to withhold a motor response.^24^ Action withholding was determined by the percentage of no-go commission errors. Commission errors were measured as the percentage of errors made where the participant did not inhibit their response on the no-go and stop trials.

Action cancellation is defined as the ability to inhibit an already ongoing motor response.^24^ Action cancellation was measured with the stop signal RT (SSRT) and percentage of stop commission errors. The SSRT was estimated using the integration method by varying the stop-signal delay (SSD) and computing the SSRT (ms) scores by subtracting the mean SSD from the mean percent RT, replacing go-omission errors with the maximum RT to compensate for lacking responses, producing the most reliable and least biased nonparametric estimates.^25^ For further information on the HRI task, please see the paper by Sebastian et al.^11^

### Statistical analysis

The program G*Power version 3.1 was used to estimate the statistical power of the study, assuming a medium effect size of 0.25, a sample size of 24 participants, and a significance level of 0.05.^26^ The power analysis indicated a power of 0.65. The analysis included covariates determined through an independent-samples *t*-test to assess education differences between groups and a Fisher–Freeman–Halton test to explore the relationship between education level and group. This was to account for the significant correlation between education and scores on behavioral measures of response inhibition found in other literature.^27^

A two-way mixed analysis of variance (ANOVA) with a repeated-measures within– between interaction was used to conduct the analysis. The presence of outliers was assessed with visual inspection of boxplots. Standardized residuals were also calculated to assess the presence of residual outliers outside the range of ±3. The analysis was performed for the following variables: (i) pre-/post-iTBS mean RT of go trials; (ii) pre-/post-cTBS mean RT of go trials; (iii) pre-/post-iTBS Simon interference effect; (iv) pre-/post-cTBS Simon interference effect; (v) pre-/post-iTBS SSRT; and (vi) pre-/post-cTBS SSRT. Assumptions of normality, homogeneity of variance, and homogeneity of covariance were checked. The dependent variables that did not follow a normal distribution underwent transformations until the normality assumption was met. An interaction effect was first investigated between time (pre/post) and group (BD/control) on the outcome variables. Next, the main effects of time and group were assessed. The analyses were further explored by examining the pairwise comparisons of group and time based on estimated marginal means with the Bonferroni correction (to account for multiple comparisons) for all possible pairs of categories. The upper bound and lower bound 95% confidence intervals (95% CIs) were also evaluated. For a statistically significant difference, *p* ≤ 0.05.

The non-normal distribution of commission error percentages, attributed to a high concentration of zeroes (a floor effect where participants made no errors, reflecting ceiling-level performance), violated the ANOVA normality assumption. Thus, an ordinal logistic regression analysis was conducted for the following variables: (i) pre-/post-iTBS no-go commission errors; (ii) pre-/post-cTBS no-go commission errors; (iii) pre-/post-iTBS stop commission errors; and (iv) pre-/post-cTBS stop commission errors. To assess any baseline differences between the BD and control groups in pre-TBS commission errors while controlling for covariates, an analysis of covariance (ANCOVA) was conducted. The assumptions of normality and homogeneity of variances were checked. For the ordinal logistic regression analysis, the proportional odds assumption was checked. The model was assessed for goodness of fit, and a likelihood ratio (omnibus) test was conducted. The difference between the pre- and post-TBS commission errors was computed, and results were placed into three categories: decreased (improved) = 2, no change (stable) = 1, and increased (worsened) = 0. The odds ratio indicated the magnitude and direction of the effect. The “no change” category captures all participants whose error count did not change—whether they remained at zero (ceiling of optimal performance) or at any other level—thereby treating preserved performance as a non-worsened outcome. Thus, a value >1 indicated that BD is associated with a higher odd of having a stable or better outcome (ie, “no change” or “improved”), and a value >0 and <1 indicated that BD is associated with a higher odd of a worsened outcome. Finally, cross-tabulations were conducted to view the frequency and percentages of BD participants and controls in each category of the commission errors to visualize any differences between groups. All analyses were conducted in IBM Statistical Package (SPSS 28, IBM Corporation, Chicago, IL).

## Results

The demographics of the sample are shown in Table 1. Education level differed between the BD and control groups (*p* = 0.03) and was therefore included as a covariate.

**Table 1.** Demographics of the Participants (*N* = 24)

	BD group (*N* = 12)	Control group (*N* = 12)
	*N* (%) or mean ± SD	*N* (%) or mean ± SD
Age (years)	29.17 ± 6.56	29.08 ± 6.80
Sex		
Female/male	10 (83.33)/ 2 (16.67)	10 (83.33)/ 2 (16.67)
Race		
White	7 (58.33)	2 (16.67)
Asian	4 (33.33)	6 (50.00)
Hispanic	1 (8.33)	2 (16.67)
Black	0	1 (8.33)
Prefer not to disclose	0	1 (8.33)
Education (years)	16.67 ± 1.88	18.50 ± 3.09
Education level		
Some college/university	2 (16.67)	3 (25.00)
College/university diploma	9 (75.00)	3 (25.00)
Graduate studies	1 (8.33)	6 (50.00)
State anxiety		
Before iTBS	35.33 ± 8.63	32.78 ± 7.40^a^
Before cTBS	34.73 ± 7.98^b^	33.44 ± 7.14^a^

*Note.* State anxiety scores were derived from the STAI. Higher scores indicate greater anxiety.

a *N* = 9 due to missing or declined to respond data.

b *N* = 11 due to missing or declined to respond data.

When controlled for education level, the group showed a significant effect (*p* = 0.01) on the pre-/post-iTBS no-go commission errors, a measure of action withholding. We found the BD group to have a strong effect on the no-go commission errors (Exp(B) = 31.92; 95% CI = 2.26, 450.52), indicating participants in the BD group made fewer no-go commission errors following iTBS. The odds ratio showed that the BD group had 32 times higher odds of having a stable or better outcome (“no change” or “improved”) following iTBS. Descriptive statistics are presented in Table 2. This was further investigated in the cross-tabulation (Table 3), where the BD group had 50% of their participants with improved no-go scores post iTMS compared to controls who had 16.7% of their participants with improved scores.

**Table 2.** Descriptive Statistics of iTBS No-Go Commission Errors by Group and Time Point

		Mean (SD)	Median
Bipolar	Pre-iTBS	7.92 (10.55)	5
(*N* = 12)	Post-iTBS	5.00 (8.26)	0
Controls	Pre-iTBS	2.92 (4.90)	0
(*N* = 12)	Post-iTBS	3.33 (5.77)	0

**Table 3.** Cross-Tabulation of iTBS No-Go Commission Errors

		Performed worse	No change	Improved
Bipolar	Count	2	4	6
	% Within group	16.7%	33.3%	50.0%
Controls	Count	3-	7	2
	% Within group	25.0%	58.3%	16.7%

The ANCOVA revealed no significant differences between groups in pre-TBS no-go commission errors (*F*(1, 22) = 1.968, *p* = 0.175), indicating that the observed effects following iTBS are not attributable to preexisting disparities in no-go errors between the groups.

There was no significant effect of the group on the pre-/post-cTBS no-go commission errors. There were also no other significant interactions or main effects for the following outcome variables pre/post iTBS or cTBS, such as mean RT, Simon interference effect, SSRT, and stop commission errors. Therefore, the results showed the BD group, but not the control group, had significantly higher odds of improving on measures of action withholding following iTBS, but not after cTBS.

Participants’ comfort ratings immediately following each TBS session are summarized in Table 4. In terms of next-day adverse effects, of the 12 BD participants, 4 reported adverse effects after iTBS (1 mild headache, 2 mild–moderate fatigue, 1 mild light-headedness, 1 moderate agitation, 1 moderate neck pain, and 1 sleep disturbance). After cTBS, 6 BD participants reported adverse effects (3 mild–moderate headaches, 3 moderate fatigues, 1 moderate neck pain, and 1 sleep disturbance). Of the 9 controls with complete data (3 missing due to decline to respond or missing responses), none reported adverse effects the morning after iTBS, while 3 reported adverse effects after cTBS (1 mild headache, 1 sleep disturbance, and 1 mild discomfort).

**Table 4.** Adverse Effects Reported Immediately after TBS (Ratings: 1 = Not at all; 10 = Extremely)

	iTBS		cTBS	
	BD group (*N* = 12)	Control group (*N* = 9)^a^	BD group (*N* = 12)	Control group (*N* = 9)^a^
	*N* reported (mean ± SD)	*N* reported (mean ± SD)	*N* reported (mean ± SD)	*N* reported (mean ± SD)
Pain	6 (4.17 ± 1.94)	5 (2.40 ± 0.55)	3 (4.00 ± 1.53)	2 (2.50 ± 0.71)
Tingling	10 (4.30 ± 1.42)	7 (4.29 ± 2.06)	4 (3.25 ± 1.29)	1 (2.00 ± 0.00)
Burning	0	0	1 (3.00 ± 0.00)	0
Fatigue	4 (4.00 ± 1.22)	3 (3.33 ± 2.31)	4 (5.00 ± 2.16)	2 (4.50 ± 1.50)
Nervousness	5 (3.20 ± 1.64)	8 (3.00 ± 0.93)	2 (2.50 ± 0.71)	2 (2.50 ± 0.71)
Disturbed concentration	3 (3.00 ± 1.00)	1 (4.00 ± 0.00)	3 (3.33 ± 1.00)	1 (2.00 ± 0.00)
Disturbed visual perception	2 (6.50 ± 3.54)	2 (3.50 ± 0.71)	2 (3.50 ± 1.50)	1 (4.00 ± 0.00)
Headache	4 (4.75 ± 1.71)	1 (4.00 ± 0.00)	5 (3.60 ± 1.87)	0
Simulation uncomfortable	8 (4.38 ± 1.74)	5 (3.80 ± 1.72)	6 (3.50 ± 1.77)	4 (2.25 ± 0.50)

a *N* = 9 due to missing or declined to respond data.

## Discussion

The present study investigated the effects of increasing cortical excitability (iTBS) and decreasing cortical excitability (cTBS) of the rIFG on the performance scores of a novel computerized task measuring three subcomponents of response inhibition in BD and controls. The response inhibition subcomponent “action withholding”—measured by no-go commission errors—was significantly improved in the HRI task following iTBS for the BD group. There were no other significant effects observed.

A possible explanation for why increasing the cortical excitability of the rIFG only had a significant effect on action withholding and not interference inhibition or action cancellation is that the three subcomponents are required at different time points in the programming and generation of a response.^11^ In the cognitive process of response inhibition, action withholding is positioned in between interference inhibition and action cancellation. Interference inhibition is an early subcomponent because it requires the inhibition of response tendencies that are involuntarily activated by incongruent stimuli, which are thought to arise before response initiation.^9^ In contrast, action cancellation is considered a late subcomponent because it assesses the inhibition of an ongoing response.^11^ Action withholding is an intermediate subcomponent of response inhibition because it comprises aspects of both action selection and inhibitory action.^11^ Therefore, the rIFG may be more involved in the “intermediate stage” of the response inhibition process in individuals with BD.

Furthermore, interference inhibition and action cancellation both involve a spatial response selection in addition to a movement initiation, while action withholding only requires participants to decide between responding and withholding a response. Interference inhibition and action cancellation also have increased pre-supplemental motor area (pre SMA) activation relative to action withholding in the HRI task.^11^ Therefore, the pre-SMA region might be more involved in downstream movement executions (eg, initiating and retracting the movement in action cancellation and performing a button press in interference inhibition).^11^ In contrast, stimulation of the rIFG in BD might be more involved in withholding a response, and thus only improves the action withholding subcomponent. Future studies should consider investigating the differences between stimulating the rIFG and the pre-SMA, their effects on the three response inhibition subcomponents, and how each of these subcomponents may relate to behavioral and/or treatment outcomes.

In addition, recent theories of TMS effects suggest that short-term cortical reorganization may occur, triggering within-network compensation and potentially canceling out any TMS effects over the rIFG.^28^ This could explain why no changes were observed following cTBS; decreasing cortical excitability may prompt another brain area to upregulate, compensating for the disruption caused by cTBS.^28^ Future research is needed to further explore this hypothesis.

### Limitations

There were some limitations in this study. First, the power of the study was determined to be 0.65, which is lower than the typical desired power of ≥0.80.^29^ This reduced the chances of detecting a true effect and, therefore, the model was more likely to produce false negative results (Type II errors). Future investigations of iTBS in BD with a larger sample size would increase the power of the study and may therefore be able to detect more significant differences in the analyses.

Recruitment challenges also resulted in a predominantly female sample of BD participants (83.33% females and 16.67% males), with sex-matched controls to eliminate group differences. Globally, the male-to-female prevalence ratio for BD is 0.8.^30^ The gender imbalance in our sample does not represent the proportion of males and females with BD in the general population and may therefore influence the study’s external validity and restrict the extent to which the conclusions can be applied to the wider population.

The final limitation to our study was the lack of double-blinding of the type of TBS administered. iTBS was delivered during Session 2, and cTBS was delivered during Session 3. While the participants were unaware of the specific treatment order, the researchers were aware of the assigned protocols. Nonetheless, efforts were made to ensure Sessions 2 and 3 were conducted uniformly, differing only in the type of TBS administered. Furthermore, there was sufficient time between sessions, ranging from 24 h to 9 d apart, together with the brief aftereffects of a single rTMS session (<70 min).^31^ This ensured the effect from the first session did not affect the second session. A placebo condition was not included to blind participants in the study, as the primary goal was to compare HRI performance scores pre and post each TBS session.

## Conclusion

Our findings indicated that iTBS of the rIFG affected a specific subcomponent of response inhibition, ‘action withholding’, in individuals with BD that was not seen in controls. Overall, the exploratory analyses offered an initial evaluation of the effects of neuromodulation on response inhibition in BD, demonstrating the potential efficacy of this novel intervention. However, larger studies investigating rTMS of the rIFG are needed to draw definitive conclusions from our findings. In summary, this proof-of-concept study represented an important early step toward developing a novel therapeutic approach tailored to address the difficulties in impulse control often associated with BD to ultimately improve treatment outcomes.

## Data Availability

The data that support the findings of this study are available from the corresponding author upon reasonable request.
